# Material Removal Mechanism in Photocatalytic−Assisted Jet Electrochemical Machining of SiC_p_/Al

**DOI:** 10.3390/mi13091482

**Published:** 2022-09-06

**Authors:** Feng Wang, Jing Zhou, Siyi Wu, Xiaoming Kang, Lin Gu, Wansheng Zhao

**Affiliations:** 1 School of Mechanical Engineering, Shanghai Jiao Tong University, Shanghai 200240, China; 2 State Key Laboratory of Mechanical System and Vibration, Shanghai 200240, China

**Keywords:** silicon carbide particle reinforced aluminum, photocatalytic−assisted jet electrochemical machining (PAJECM), hybrid machining

## Abstract

Silicon carbide particle reinforced aluminum matrix (SiC_p_/Al) composites are increasingly used in high−end industries because of their superior comprehensive material properties. However, their advanced properties also create severe challenges for traditional processing technologies. A new hybrid machining method named photocatalytic−assisted jet electrochemical machining (PAJECM) is proposed to improve the machining capability by synchronously removing the metal aluminum matrix and the SiC particles. Comparative experiments were carried out on whether photocatalysis was added. The results show that after photocatalytic−assisted jet electrochemical machining, the height of SiC particles’ extrusion on the surface is significantly reduced. Compared with jet electrochemical machining (JECM) without photocatalysis at the same processing voltage, the surface roughness value is reduced, and the processing quality is improved. In PAJECM, the aluminum matrix is removed by electrochemical anodic dissolution, while the SiC particles generate a SiO_2_ reaction layer through photocatalysis, and the TiO_2_ abrasive flow’s mechanical action repeatedly removes the reaction layer. The electrochemical polarization curves and energy diffraction spectroscopy elemental analysis confirmed the material removal mechanism of PAJECM. Based on analyzing the phenomenon of material removal in detail, a qualitative model of the PAJECM material removal mechanism is established. This study provides valuable insights into the material removal mechanism in photocatalytic and jet electrochemical machining composite processes.

## 1. Introduction

Silicon carbide particle reinforced aluminum matrix (SiC_p_/Al) composites are widely used in aerospace, precision instruments, and electronic industries due to their excellent mechanical and physical properties [1], such as high specific strength and modulus, good thermal stability, and wear and corrosion resistance. For example, they are applied as the structural material in precision parts for inertial navigation systems, laser gyroscopes, and optical mirrors. However, the characteristics of high hardness, high brittleness, and high strength [2] of SiC ceramic particles make SiC_p_/Al composites belong to difficult−to−cut materials. Unfortunately, these precision parts contain many microstructures that should be precisely machined or polished [3]. When processed by traditional cutting methods [4], they are prone to produce chip collapse, workpiece edge damage, tool wear, and other phenomena [5,6]. When machining with thermal effects, such as electric discharge machining (EDM) [7] and laser machining [8], they are likely to form recast layers, microcracks, and heat−affected zones on the surface, which also affect the performance of the workpiece [9].

Unlike traditional cutting and thermal processes, electrochemical machining removes materials depending on anodic dissolution. During the machining process, there is no tool wear, residual stress, or heat−affected zone [10]. Jet electrochemical machining (JECM) is one of the specific processes of electrochemical machining (ECM). In addition to the advantages of the ECM process, jet ECM shows excellent process flexibility and a stable machining process [11]. In JECM, the electrolyte is injected into the workpiece anode through the nozzle cathode to form the electrolyte jet. The thin electrolyte layer around the jet has a restraining effect on the electric field distribution, so the machining current is mainly concentrated on the workpiece surface impacted by the jet [12]. Using the displacement platform to drive the cathode of the nozzle to move according to the preset path can realize the rapid and precise manufacturing of some complex structures on the workpiece surface [13]. Therefore, JECM has great processing potential in SiC_p_/Al composites [14], and scholars have carried out some exploratory studies [15].

Hackert et al. studied JECM processing of SiC_p_/Al with a SiC particle diameter of less than 1μm and a volume fraction of 10% [16]. They found that the feed rate increases with the current density within a specific range. The machined surface roughness value significantly decreases at a higher current density in a specific range [14]. When the current density increases over 10 A/cm², the roughness no longer decreases because SiC particles cannot be processed in NaNO_3_ neutral electrolytes [17]. Hackert et al. also studied the dissolution properties of three neutral electrolyte solutions for jet electrochemical machining of SiC_p_/Al at a voltage of 60 V, and found that using NaNO_3_ electrolytes can produce a better−machined surface quality [18]. Ao et al. measured the polarization curves in NaNO_3_ and NaCl electrolytes and compared the dissolution characteristics of SiC_p_/Al in the two electrolytes. They found that increasing the electrolyte concentration and processing voltage improves the material removal rate [19]. However, it leads to a rougher processing surface. At a voltage of 40 V, the minimum surface roughness value is Ra 3.8 μm. Liu et al. analyzed the material removal mechanism of SiC_p_/Al by jet electrochemical machining [20]. They found that the aluminum matrix near the SiC particles is first removed by anodic dissolution. The SiC particles are washed off by the jet. The larger the particle size of SiC particles, the rougher the surface, and a surface roughness of Ra 3~5 μm can be obtained. Liu et al. studied the abrasive−assisted electrochemical jet machining of SiC_p_/Al [21]. High−speed abrasives can destroy the oxide layer generated on the surface of the workpiece and remove a part of the material to obtain a higher material removal rate [22].

Because SiC particles are materials with poor electrical conductivity [23], traditional electrochemical machining cannot effectively remove SiC particles, leaving protrusions or dimples that affect the quality of the machined surface. This paper proposes a new SiC_p_/Al machining method named photocatalytic−assisted jet electrochemical machining (PAJECM), which can simultaneously remove the metal aluminum matrix and SiC particles to produce a higher surface quality in the local processing area. It has good application potential in the fields of aerospace, optical precision instruments, and electronic packaging. In order to further clarify the process characteristics of PAJECM, the material removal mechanism in the PAJECM process was studied in detail.

## 2. Principle of PAJECM Process

**Figure 1:**
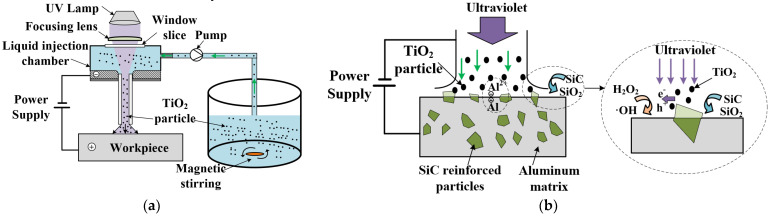
Photocatalytic−assisted jet electrochemical machining principle of composite SiC_p_/Al (**a**) Schematic diagram of PAJECM; (**b**) Processing principle.

A schematic diagram of photocatalytic−assisted jet electrochemical machining (PAJECM) is shown in Figure 1a. The electrolyte solution comprises NaNO_3_, H_2_O_2_, and TiO_2_ abrasive particles, forming a uniform suspension under magnetic stirring. The PAJECM achieves material removal through the combined action of electrochemical anodic dissolution and TiO_2_ abrasive jet removal of the SiC chemical reaction layer. The material removal mechanism of PAJECM processing SiC_p_/Al is more complicated than that of jet electrochemical machining. On the one hand, SiC particles are materials with poor conductivity and cannot be removed by electrochemical anodic dissolution. On the other hand, passivation films easily form on the surface of the aluminum substrate in NaNO_3_ electrolyte. The passivation film will hinder the electrochemical reaction but also reduce stray corrosion.

The processing principle of PAJECM is shown in Figure 1b. TiO_2_ particles can generate electron−hole pairs under ultraviolet irradiation and react with H_2_O_2_ to generate hydroxyl radicals with high oxidative ability •OH [24]. The •OH generated by the photocatalytic reaction chemically reacts with SiC to form a SiO_2_ reaction layer with a hardness much lower than that of SiC, which is easily removed mechanically. The reaction process is shown in Equations (1)−(4) [25,26]. Under the impact pressure of the jet, the TiO_2_ abrasive flow removes the SiO_2_ reaction layer, exposing the new SiC surface and continuously chemically reacting with •OH to form the SiO_2_ reaction layer, realizing the material removal of SiC particles.

(1) $${\mathrm{TiO}}_{2}+hv\to h^{+}+e^{-}$$

(2) $$e^{-}+H_{2}O_{2}\to •\mathrm{OH}+{\mathrm{OH}}^{-}$$

(3) $$h^{+}+H_{2}O\to H^{+}+•\mathrm{OH}$$

(4) $$\mathrm{SiC}+4•\mathrm{OH}+O_{2}\to {\mathrm{SiO}}_{2}+{\mathrm{CO}}_{2}+2H_{2}O$$

Therefore, the removal method of SiC_p_/Al material by PAJECM should include two processes. One process is that the aluminum matrix is removed by electrochemical anodic dissolution. Another process is to repeatedly and alternately remove SiC−reinforced particles through photocatalytic reaction and mechanical action of abrasive flow.

## 3. Experimental Setup

**Figure 2:**
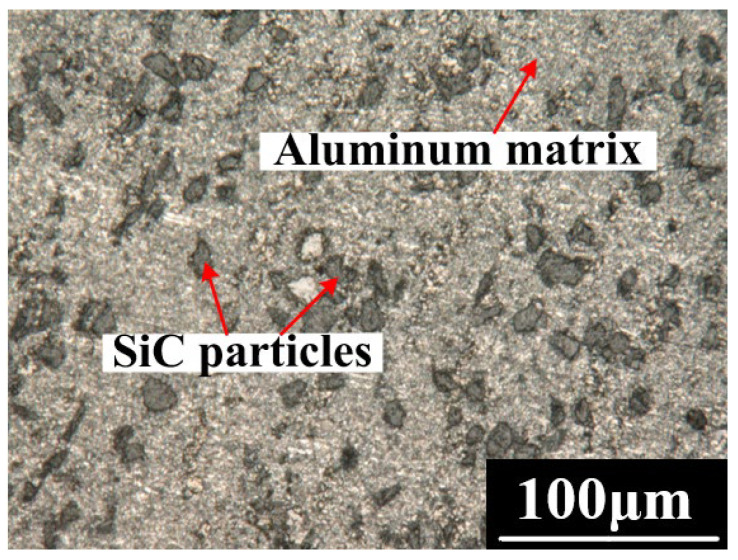
Microstructure of SiC_p_/Al composites.

**Figure 3:**
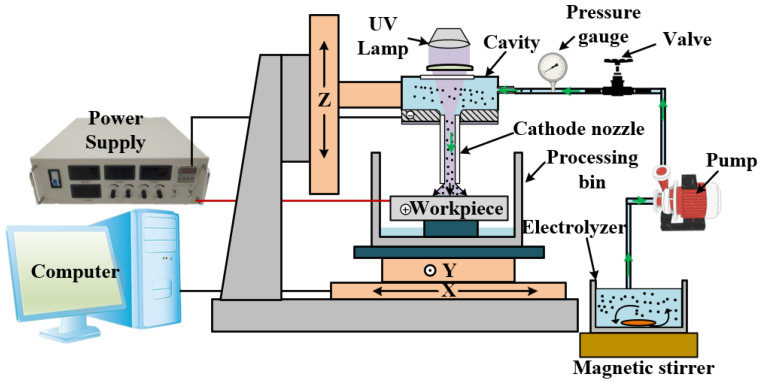
Schematic diagram of the PAJECM experimental setup.

SiC_p_/Al with a SiC volume fraction of 20% was used in the experiment. The size of the SiC reinforced particles was 7~15 μm, and the size of the test piece was 30 mm × 30 mm × 3 mm. Figure 2 shows the microstructure of SiC_p_/Al. The experimental device consisted of an XYZ platform, cathode nozzle, pump, power supply, UV lamp, and magnetic stirrer, as shown in Figure 3. The cathode nozzle was made of a stainless−steel capillary with an outer diameter of 1.0 mm and inner diameter of 0.8 mm. The metering pump drew the mixed abrasive electrolyte through the cathode nozzle to form a jet with a diameter of about 0.8 mm. The power supply outputs 10–60 V DC voltage, the cathode was connected to the metal capillary nozzle, and the anode was connected to the workpiece. The SiC_p_/Al specimen was installed on the XY platform, which could be moved in a two−dimensional direction during the experiment. The cathode nozzle was installed on the Z−axis, and the inter−electrode gap could be adjusted by electrical sensing. 

Table 1 shows the various process conditions used in the experiment. The machining gap was set to a constant value of 0.3 mm. Traditional JECM uses NaNO_3_ with a mass fraction of 15% as the electrolyte working fluid. PAJECM uses a suspension mixed with 15% NaNO_3_, 3% volume fraction H_2_O_2,_ and 4 g/L TiO_2_ abrasive particles as the electrolyte working fluid. The magnetic stirrer was working to make the TiO_2_ abrasive particles evenly distributed in the electrolyte solution during processing. The electrolyte solution was irradiated with an ultraviolet LED lamp, the illumination intensity was 1500 mW/cm^2^, the electrolyte flow rate was 200 mL/min, and the processing time was 30 s. 

**Figure 4:**
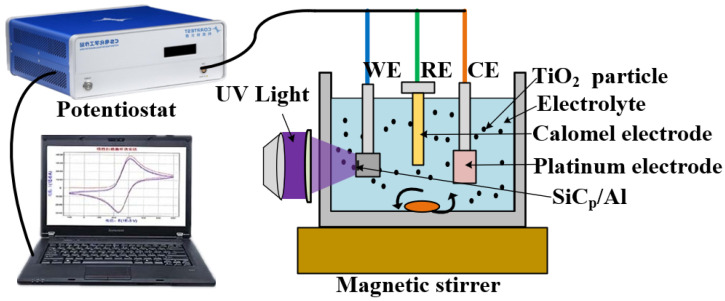
Schematic diagram of the electrochemical measurement device.

As shown in Figure 4, a potentiostat was used to measure the electrochemical experiment. In this three−electrode system, a platinum electrode with an area of 1.5 cm^2^ (1 cm × 1.5 cm) was used as the counter electrode (CE), and a saturated calomel electrode (SCE) was used as the reference electrode (RE). The SiC_p_/Al sample was attached to the working electrode (WE) holder with a conductive adhesive and wrapped with Teflon insulating tape. The surface area of the SiC_p_/Al exposed to the electrolyte was 4 mm^2^. Before each electrochemical measurement, the samples were sequentially polished with different grit−sized sandpaper to remove the oxide layer, then washed with ethanol and dried. We used 100 mL of electrolyte for each experiment, and we changed the electrolyte after each electrochemical measurement. After soaking the sample for 10 min, we measured the open circuit potential (OCP) and waited for half an hour for the open circuit potential to stabilize. Dynamic potential polarization curves (Tafel plots) were obtained at a scan rate of 1 mV/s over the specified dynamic potential range (from OCP drop of 500 mV to OCP rise of 500 mV). Potential scanning was performed in the range of 0 to 10 V, and the dissolution characteristic curve of decomposition voltage was obtained at a scanning speed of 10 mV/s.

Each processing experiment was repeated three times, and the result was the average value of the three measurements. The differences in surface morphology, material removal rate, and surface roughness between traditional JECM and PAJECM at different processing voltages were studied.

## 4. Experimental Results

**Figure 5:**
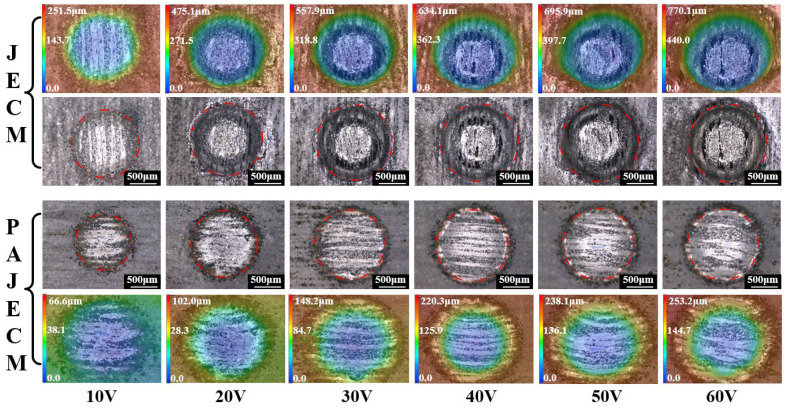
Comparison of surface morphologies processed by JECM and PAJECM at different voltages.

Figure 5 shows the surface morphology comparison between JECM and PAJECM at different voltages from 10 to 60 V, and a processing time of 30 s. The black−and−white images represent two−dimensional optical images, and the red dotted line is the outline boundary of the machining dimple. The three−dimensional depth map is represented by the color map, and the white numbers in the color map are the depth scale. It can be seen that the two processes have apparent differences in machining morphology. At the PAJECM processing voltage of 10 to 30 V, the roundness of the machined contour of the two−dimensional image and the consistency of the image surface are poor. It indicates that the removal uniformity of the workpiece material is poor, possibly due to the low machining voltage and short machining time, resulting in insufficient material removal. At the voltage of 40~60 V, the image surface consistency of the PAJECM processing dimple is better than that of JECM. The distribution of SiC particles in the central area can be clearly seen, which indicates that PAJECM−processed SiC_p_/Al composites can obtain better results—finished surface quality. Compared with PAJECM, the stray corrosion at the edge of the dimples processed by JECM is more pronounced. The reason is that solid oxidizing substances, •OH, are generated during the processing of PAJECM. A passivation layer easily forms on the processed surface, which is destroyed and removed under the impact of the abrasive jet. Therefore, the material removal area of PAJECM is concentrated inside the jet. The electrolyte solution used in JECM is only NaNO_3_ solute, and the material in the outer area of the jet is electrochemically dissolved.

**Figure 6:**
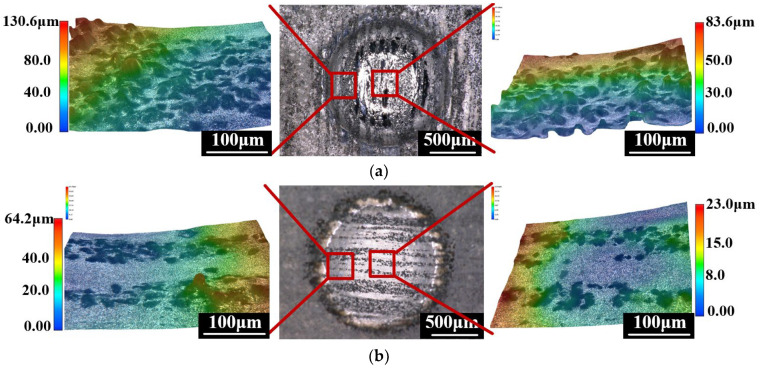
Comparison of microstructures at the processing voltage of 40 V: (**a**) JECM; (**b**) PAJECM.

Figure 6 shows a comparison of the microscopic morphology at the processing voltage of 40 V. Because SiC particles are materials with poor conductivity, they cannot be removed by anodic dissolution. As shown in Figure 6a, there is a large number of SiC particles on the microscopic surface after JECM processing. However, after PAJECM processing, the microscopic surface of SiC_p_/Al is relatively flat, as shown in Figure 6b, indicating that the raised SiC particles are effectively removed. That is, PAJECM improves the surface quality of SiC_p_/Al processing.

**Figure 7:**
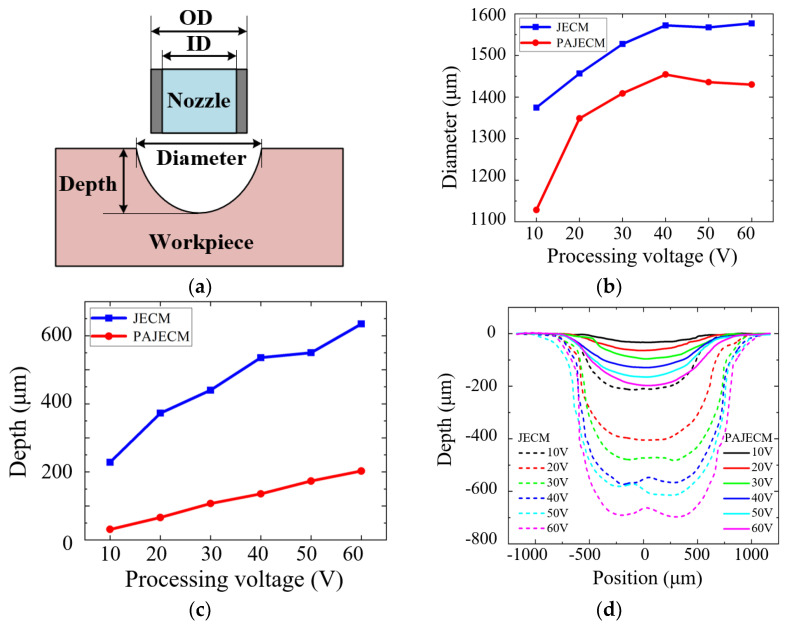
Diameter and depth of dimples processed by different processes: (**a**) schematic diagram of dimples; (**b**) dimple diameter; (**c**) dimple depth; (**d**) cross−section profile.

Figure 7 shows a comparison of the experimental results of the dimple diameter and dimple depth processed by JECM and PAJECM at different voltages. As shown in Figure 7b, the dimple diameter gradually increases with the increase in processing voltage. At the same processing voltage, the diameter of the dimples processed by PAJECM is significantly smaller than that of JECM, indicating that photocatalytic assistance reduces stray corrosion and improves processing accuracy. However, the depth of the dimples processed by PAJECM is also significantly reduced, as shown in Figure 7c. Due to the strong oxidation •OH generated during PAJECM processing, a dense oxide layer is formed on the aluminum substrate’s surface, reducing the electrochemical anode’s dissolution rate. Figure 7d depicts a cross−sectional profile comparison. Because a stainless−steel capillary tube is used as a nozzle, it differs from the gemstone nozzle used in conventional JECM. The strongest electric field is concentrated between the inner and outer diameters of the stainless−steel capillary. In JECM, when the voltage is large, the current density distribution is uneven and prominent, and the current density at the edge is greater than that at the center, thus forming an island inside the dimple.

**Figure 8:**
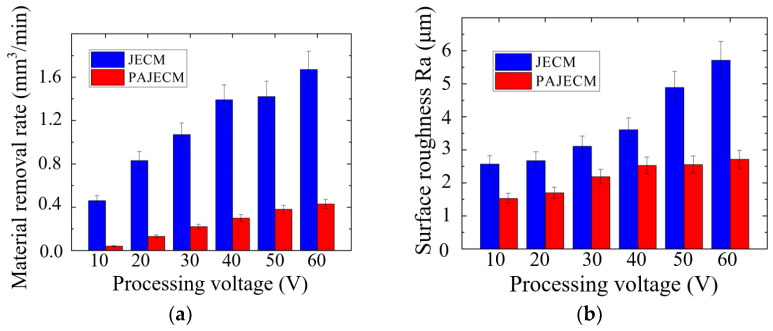
Comparison of experimental results at different processing voltages: (**a**) material removal rate; (**b**) surface roughness Ra.

As shown in Figure 8a, the material removal rate of PAJECM is significantly lower than that of JECM. PAJECM can be used for finishing or small allowance material removal. As shown in Figure 8b, compared with JECM, the surface roughness value Ra of PAJECM decreases from Ra 2.5 to Ra 1.5 μm when the processing voltage is 10 V and from Ra 5.7 to Ra 2.7 μm when the processing voltage is 60 V. At the same processing voltage, the surface roughness of the two processes greatly varies, and PAJECM significantly reduces the surface roughness value. During PAJECM, the surface of the SiC particles exposed in the mixed electrolyte react with •OH to form a SiO_2_ layer with lower hardness. The TiO_2_ abrasive flow repeatedly removes the layer, and the generated •OH further enhances the passivation of the electrolyte, resulting in a significant improvement in the surface quality.

## 5. Analysis and Discussion

### 5.1. Electrochemical Characterization Analysis

**Figure 9:**
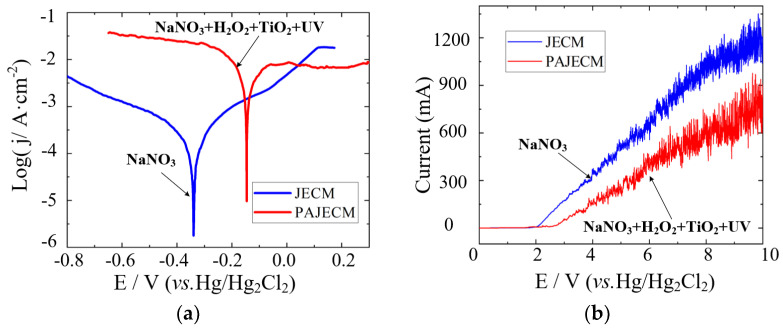
Comparison of electrochemical polarization curves at different processing conditions: (**a**) Tafel curve; (**b**) decomposition voltage curve.

As shown in Figure 9a, in the NaNO_3_ electrolyte used in JECM, the self−corrosion potential of SiC_p_/Al composites is maintained at around −0.34 V. In the composite electrolyte used in PAJECM and with the assistance of ultraviolet light, the self−corrosion potential of the SiC_p_/Al composite material is maintained at around −0.15 V. The corrosion resistance of SiC_p_/Al in the PAJECM electrolyte is significantly enhanced, indicating that the composite electrolyte’s passivation performance is improved. The decomposition voltage curve is shown in Figure 9b. The decomposition voltage in the NaNO_3_ solution is 2.1 V, indicating that the SiC_p_/Al composite is in the passivation zone before the electrode potential is 2.1 V. Its current density remains unchanged in a minimal range with the increase in the inter−electrode voltage. The dissolution rate during electrolysis in this area is very low or even insoluble. After that, with the continuous increase in the potential, the current density on the material’s surface gradually begins to increase, indicating that the passivation film was partially dissolved and peeled off. The material gradually enters the over−passivation region, thus ensuring the continued dissolution of the SiC_p_/Al composite material. With the aid of ultraviolet light, the decomposition voltage of SiC_p_/Al in the composite electrolyte is about 2.7 V. Increasing the decomposition voltage can effectively reduce the stray corrosion of workpiece materials in ECM and improve machining accuracy. The electrochemical polarization curves showed that robust oxides are produced during PAJECM processing, which increased the self−corrosion potential and decomposition voltage of SiC_p_/Al.

**Figure 10:**
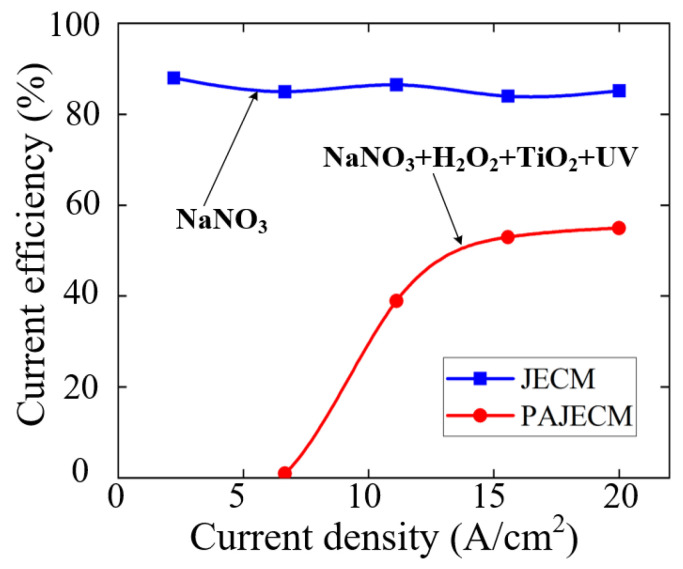
Comparison of current efficiency curves.

Figure 10 shows the current efficiencies of JECM and PAJECM at different current densities. In JECM, the current efficiency is much higher than in PAJECM, and severe stray corrosion occurs even at very low current densities. In PAJECM, however, anodic dissolution can be completely eliminated at small current densities, which favors the localization of anodic dissolution. When the current density exceeds 6.7 A/cm^2^, the current efficiency rapidly increases with the increase in the current density. The higher cut−off current density indicates that PAJECM is beneficial in reducing stray corrosion and improving machining accuracy.

### 5.2. Elemental Energy Spectrum Analysis

**Figure 11:**
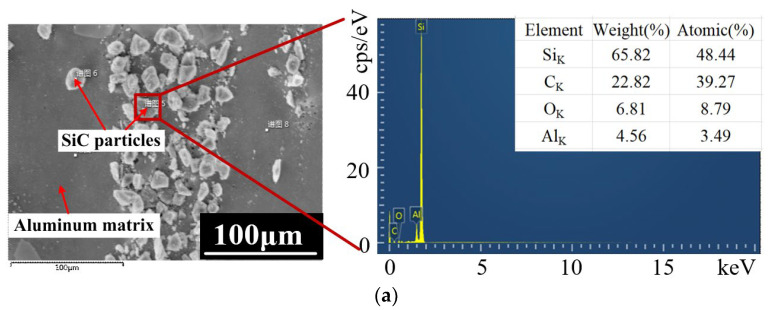

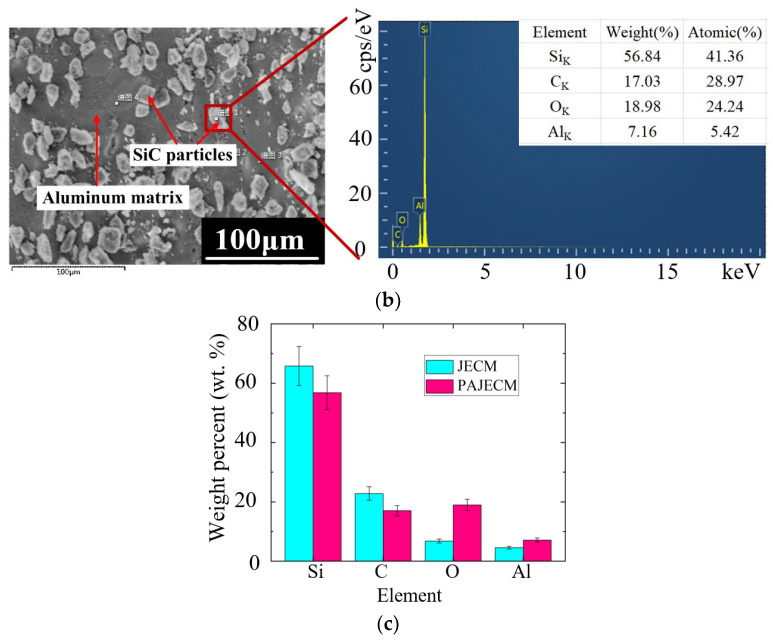
Atomic mass percentage of EDS point scan analysis of the machined surface at a voltage of 40 V: (**a**) JECM; (**b**) PAJECM; (**c**) element weight percentage comparison.

As shown in Figure 11, the energy spectrum measurement results of the surface of SiC particles after JECM and PAJECM processing at a voltage of 40 V were analyzed using the EDS point. The processed specimens were ultrasonically cleaned with ethanol and distilled water, dried with compressed inert gas, and placed in a sealed bag to prevent surface oxidation and oil stains from affecting the measurement results of surface elements. The weight percentages of C and O elements on the surface of SiC particles after JECM treatment were 39.27% and 8.79%, respectively. While the weight percentage of C elements on the surface of SiC particles after PAJECM treatment decreased to 17.03%, the weight percentage of O elements increased to 18.98%. The results showed that oxides formed on the surface of SiC particles after PAJECM processing.

**Figure 12:**
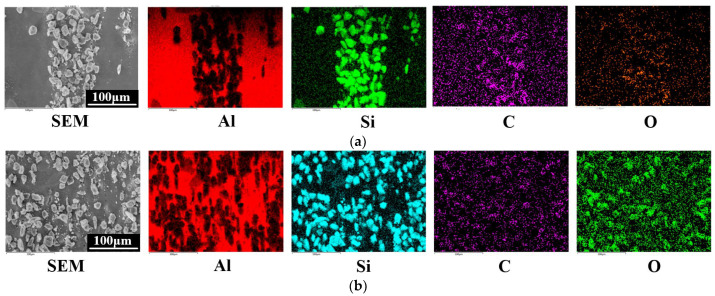
The energy spectrum of EDS element distribution on the machined surface at a voltage of 40 V: (**a**) JECM; (**b**) PAJECM.

In Figure 12, the EDS element distribution diagram shows that the distribution of C elements after JECM processing followed the distribution of Si elements, and the distribution of O elements was relatively uniform; the distribution of O elements after PAJECM processing was the same as that of Si elements, indicating that the oxide containing Si was formed. According to the value of atomic mass percentage, we speculated that the product was SiO_2_. Therefore, the chemical reaction Equations (1)−(4) were verified, and the •OH generated by the photocatalytic reaction chemically reacted with SiC to form a low−hardness SiO_2_ reaction layer.

### 5.3. Discussion

**Figure 13:**
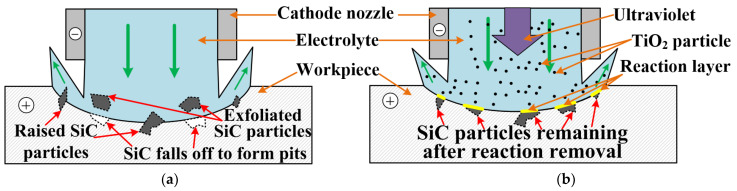
Schematic diagram of material removal mechanism in the ideal process: (**a**) JECM; (**b**) PAJECM.

Based on the experimental results discussed, a qualitative model was developed to illustrate the material removal mechanism of the PAJECM process, as shown in Figure 13. In the JECM process, many SiC particles are irregularly exposed from the matrix material with the aluminum matrix’s dissolution. SiC particles with poor conductivity cannot be removed and gradually bulge or fall off to form a rough machined surface, as shown in Figure 13 a. During PAJECM processing, TiO_2_ particles generate electron−hole pairs under ultraviolet irradiation and react with H_2_O_2_ through photocatalysis to generate •OH with high oxidation. The •OH reacts with SiC to form the SiO_2_ oxide layer with low hardness and adhesion. Under the impact of high−speed TiO_2_ abrasive flow, the reaction layer is removed, exposing a new SiC surface, and constantly reacts with •OH, repeatedly and alternately. The removal of SiC particles from materials with poor conductivity is realized. Therefore, PAJECM can synchronously remove the aluminum matrix and SiC particles, and a relatively flat machined surface is finally produced.

Therefore, as shown in Figure 13, PAJECM can achieve high−quality SiC_p_/Al processing under the combined action of the aluminum matrix being removed by electrochemical anodic dissolution and repeatedly removing the reaction layer SiO_2_ generated through photocatalytic SiC by TiO_2_ abrasive flow.

## 6. Conclusions

A new method named photocatalytic−assisted jet electrochemical machining (PAJECM) of SiC_p_/Al was designed, which can simultaneously remove the aluminum matrix and SiC particles and produce a higher surface quality in the local machining area. According to the comparison of the experimental results of JECM and PAJECM at different voltages, the following conclusions could be drawn:

(1) The material removal morphology of SiC_p_/Al in PAJECM significantly changed due to the addition of photocatalysis. The observation of the machined micro surface confirmed that the SiC particles with poor conductivity are effectively removed. Compared with JECM, the surface roughness value of PAJECM decreases from Ra 2.5 to Ra 1.5 μm when the processing voltage is 10 V, and the surface roughness value decreased from Ra 5.7 to Ra 2.7 μm when the processing voltage is 60 V. PAJECM significantly improves the surface quality of SiC_p_/Al machining.
(2) By observing the material removal phenomenon, we found that the SiC_p_/Al material removal mechanism of PAJECM is significantly different from that of JECM. In PAJECM, the aluminum matrix and SiC particles are simultaneously removed. The aluminum matrix is removed by anodic dissolution. The SiC particles are removed by two steps: the hard SiC particles form a SiO_2_ layer under the photocatalytic reaction; the comparatively soft SiO_2_ layer is removed by the abrasive particle impact. In JECM, the aluminum matrix is also removed by anodic dissolution, while the SiC particles either fall off to form a pit or remain on the machined surface to form a protrusion.
(3) The material removal mechanism of PAJECM was verified by its electrochemical polarization curves and the EDS results. After adding photocatalysis, the self−corrosion potential and decomposition potential of SiC_p_/Al significantly increase, which proves that the intense oxidizing substance •OH is generated during processing. After processing, the oxygen content on the surface is obviously increased, and the distribution of oxygen and silicon is consistent, which proves the formation of Si−containing oxide SiO_2_.

## Figures and Tables

**Table 1:** Experimental conditions.

Processing Parameters	Value
Workpiece	SiC_p_/Al with 20% SiC volume fraction, thickness 3 mm
Catalyst TiO_2_ concentration	4 g/L
H_2_O_2_ Volume fraction	3%
Electrolyte type and concentration	NaNO_3_, 15%
UV light intensity	1500 mW/cm^2^, 10 W, 365 nm wavelength
Processing voltage	DC 10, 20, 30, 40, 50, 60 V
Processing time	30 s
Flow rate	200 mL/min
Stainless-steel capillary nozzle	Inner diameter (ID) 0.8 mm, outer diameter (OD) 1 mm
Inter−electrode gap	0.3 mm

## Data Availability

Data available on request due to restrictions e.g., privacy or ethics. The data presented in this study are available on request from the corresponding author. The data are not publicly available due to not being agreed upon by all co−authors.
